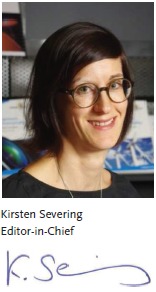# High Impact of Advanced Science

**DOI:** 10.1002/advs.201700286

**Published:** 2017-07-18

**Authors:** Kirsten Severing

**E**xactly one year ago we celebrated the first Journal Impact Factor of *Advanced Science* with the publication of an exclusive Review Special Issue. This year, there is again reason to celebrate: we are happy to announce that the impact factor of *Advanced Science* has increased by more than 50% to a value of 9.034 (*Journal Citation Reports*, Clarivate Analytics, 2017). That is an amazing result for this young journal and clearly an achievement of the strong international editorial team in combination with an excellent advisory board. Executive advisory board member Ali Khademhosseini (Harvard‐MIT, USA) recently stated: “*Advanced Science* has become a premier journal that publishes a broad spectrum of scientific breakthroughs. Thus the journal fits an important need given that such advances are increasingly interdisciplinary.”

**I**n this issue we have once again compiled a variety of overview articles that demonstrate the broad range of topics covered in the journal: From polymer science to robotics, from computer simulations of supercapacitors to photodynamic therapy, from batteries to aggregation‐induced emission – *Advanced Science* has it all.

**M**arco Rolandi and co‐workers (University of California Santa Cruz, USA) summarize their latest efforts on developing bio‐protonic devices that control H^+^ currents at the palladium hydride contact solution interface to monitor and control biological reactions. Cyrille Boyer (UNSW Sydney, Australia) highlights recent advances in the field of polymerization‐induced self‐assembly (PISA) with a focus on the visible‐light‐initiated process (photo‐PISA). Alessandro Chiolerio and Marco B. Quadrelli (Istituto Italiano di Tecnologia, Torino, Italy) provide an initial assessment of existing capabilities of liquid robotics with many future applications, such as space exploration in extreme environments, post‐disaster search, compliant wearable devices, and in vivo medical applications. Yat Li et al. (University of California Santa Cruz, USA) summarize recent advances in the area of paper‐based electrodes, with a particular focus on the methodology of fabricating these novel functional electrodes, and discuss some key challenges and opportunities in this rapidly growing field. Mingbo Wu, Jianzhang Zhao, and co‐workers (China University of Petroleum, Qingdao and University of Technology, Dalian, China) have written a review article that summarizes a variety of strategies for modulating the singlet oxygen quantum yield and thus enhancing photo­dynamic therapy efficiency. The fundamental scientific principle, structure, and possible classification of battery–supercapacitor hybrid devices are discussed in the review article by Yuanyuan Li and Jinping Liu (Wuhan University of Technology and Huazhong University of Science and Technology, Wuhan, China). Qianqian Li and Zhen Li (Wuhan University, China) elucidate the important role of molecular packing modes on aggregation‐induced‐emission (AIE) materials. In a more theoretical contribution, De‐en Jiang and co‐workers (University of California Riverside, USA) present a detailed overview of progress made in the past decade using computer simulations to understand supercapacitors. Finally, the issue contains a review article by Zhou Li (Beijing Institute of Nanoenergy and Nanosystems, Chinese Academy of Sciences, China) on biomechanical energy harvesters, nanosensors, and stimulators in the biomedical field.

**I** would like to thank all authors who have contributed their fantastic articles to this issue and I hope that you will enjoy this collection. If you are interested in contributing your own open access article that anyone can freely access, do not hesitate to contact the editorial team. We are happy to discuss any potential topic with you.

On behalf of the entire editorial team,